# Supplementary figures and images for: A Network Inference Method for Large-Scale Unsupervised Identification of Novel Drug-Drug Interactions
Source: PLoS Comput Biol. 2013 Dec 5;9(12):e1003374. doi: 10.1371/journal.pcbi.1003374 (PMC3854677; doi:10.1371/journal.pcbi.1003374)

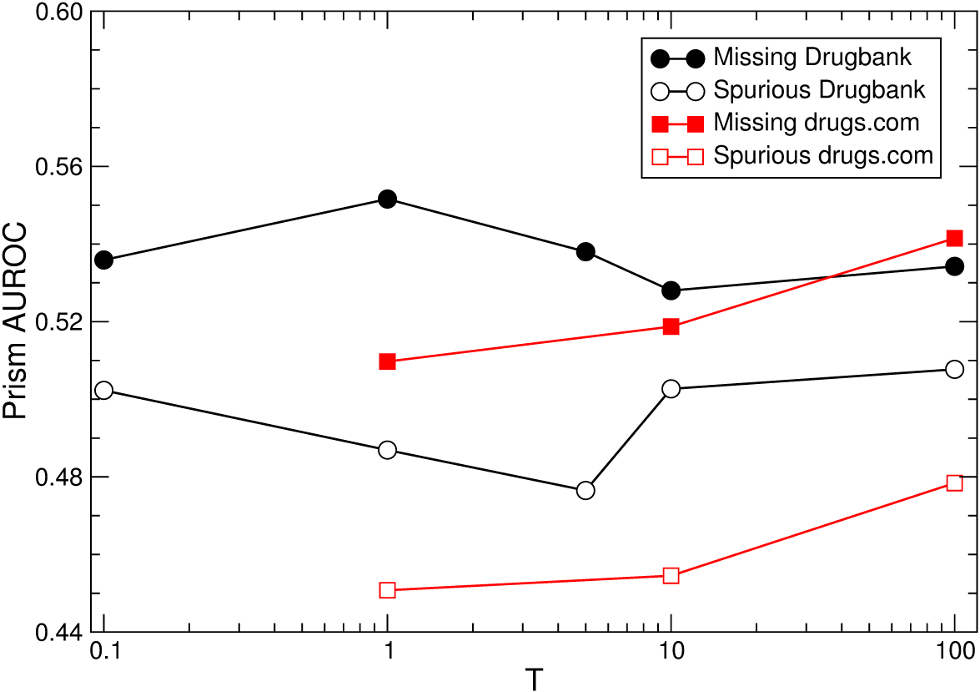

Supplement: Figure S1 — The accuracy of the Prism-based method, as measured by the AUROC, does not improve consistently and significantly by choosing values of other than , as used in the main text. (TIFF) [file pcbi.1003374.s001.tiff]

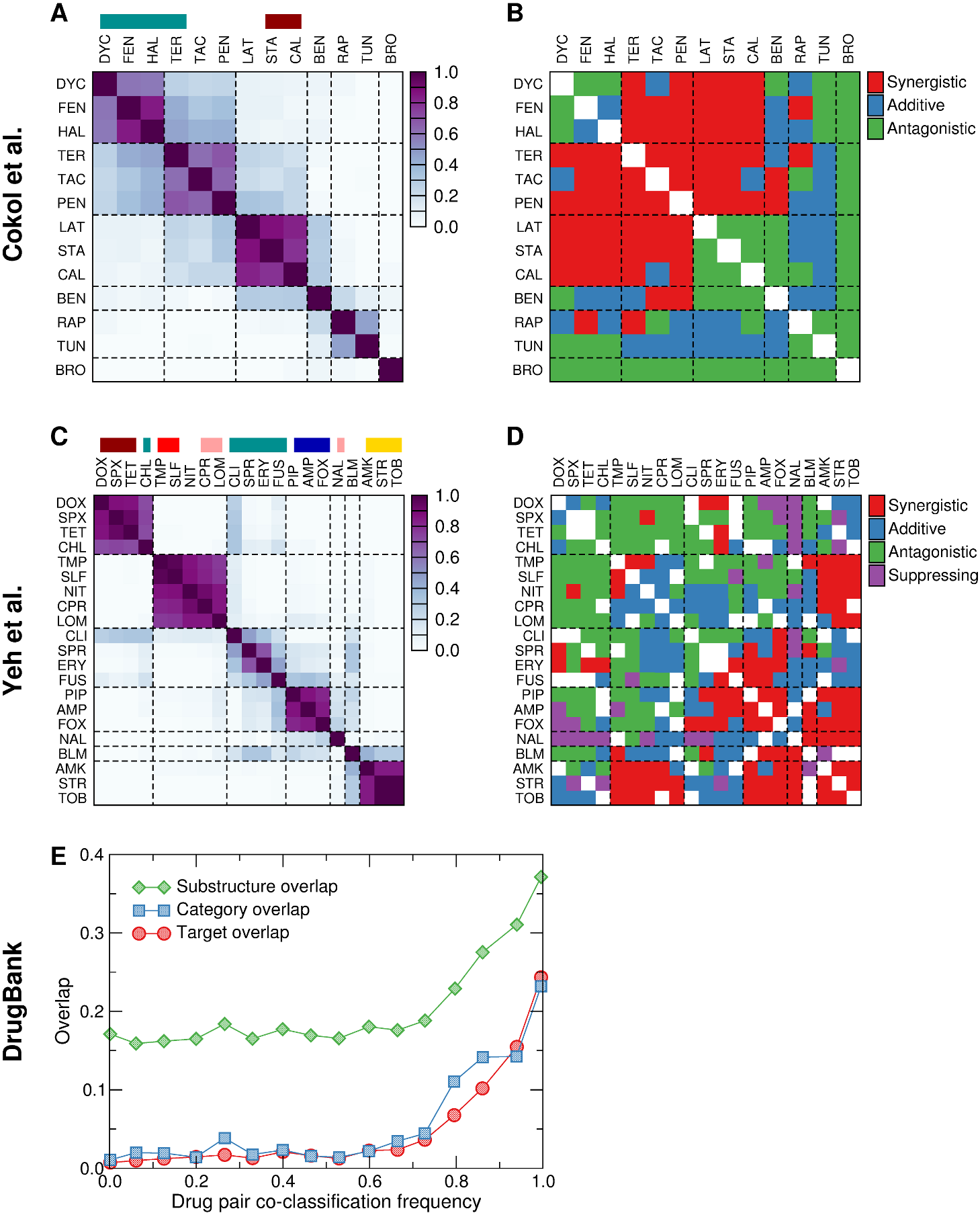

Supplement: Figure S2 — Drug groups and drug mechanisms of action from stochastic block models. For each drug pair in the Cokol et al. dataset (A–B), the Yeh et al. dataset (C–D) and the DrugBank 2012 snapshot (E), we calculate the probability that any two drugs belong to the same drug group (see Section 2). We call this probability the co-classification probability. (A) and (C) The matrix of co-classification probabilities for the Cokol et al. dataset (A), and Yeh et al. dataset (C), ordered so that large co-classification probabilities appear close to the diagonal [40]. Dashed lines are a guide to the eye. The mechanism of action of each drug is indicated by color bars on top of drug abbreviations ((A) Cyan: ergosterol metabolism; dark red: acting on serine/threonine; other drugs were intentionally selected with different targets and mechanisms of action. (C) Dark red: protein synthesis, 30S; cyan: protein synthesis, 50S; red: folic acid biosynthesis; pink: DNA gyrase; dark blue: cell wall; yellow: aminoglycoside, protein synthesis, 30S). Co-classification boxes correspond, to a large extent, to mechanisms of action. (B) and (D) The reported drug interactions show clear patterns once they are ordered according to the co-classification probability. For example in the Yeh et al. dataset, most interactions between the group and the group are synergistic. (E) We use information in DrugBank to analyze the overlap (or functional similarity) in substructure, category and target between pairs of drugs (see Section 3). We plot these quantities as a function of the co-classification probability of the corresponding drug pairs (we average over drug pairs with similar co-classification probability; error bars represent the standard error of the mean and are generally smaller than the symbols). Drugs with higher co-classification probability are significantly more likely to share substructures, categories and targets. (TIFF) [file pcbi.1003374.s002.tiff]
