# Supplementary material for: A Network Inference Method for Large-Scale Unsupervised Identification of Novel Drug-Drug Interactions
Source: PLoS Comput Biol. 2013 Dec 5;9(12):e1003374. doi: 10.1371/journal.pcbi.1003374 (PMC3854677; doi:10.1371/journal.pcbi.1003374)
Supplement: Text S1 — Sensitivity analysis for the Prism-based algorithm. Discussion on drug groups and drug mechanisms. Discussion on the estimation of the co-classification probability using stochastic block models. Analysis of drug similarities for drugs in the DrugBank. (PDF) [file pcbi.1003374.s003.pdf]

Supporting Text S1

A network inference method for large-scale unsupervised identification of novel drug-drug interactions

R. Guimerà* and M. Sales-Pardo

*To whom correspondence should be addressed. E-mail: roger.guimera@urv.cat

1 Sensitivity analysis for the Prism-based algorithm

By itself, the Prism II algorithm returns a tree of nested drug groupings. To make interaction predictions, we need to: (i) set the free parameter T; (ii) cut the tree at a certain level to get a single partition of the drugs into groups (a process that needs to be unsupervised); and (iii) given those groups, determine the probability of each type of interaction.

In the simulations reported in the main text, we set $T = 10$ to get results consistent with those reported in Ref. [1]. In Fig. (S1), we show that the results do not improve using other values of T.

2 Drug groups and drug mechanisms

As discussed above, stochastic block models only assume that: (i) there are groups of drugs; (ii) all drugs within a group interact similarly with others. Importantly, the "correct" drug groups are unknown a priori, and all that is available to the algorithms is the network of known drug interactions. Our Bayesian approach deals with this uncertainty by assigning a weight to each possible partition of the drugs into groups (Methods). Using these weights, we can estimate the

probability that any two drugs belong to the same group (without having to select any particular partition of the drugs).

Although block models do not make any assumption in this respect, it is reasonable to expect that drug groups are correlated with drug function and action mechanism. In fact, as discussed above, there is evidence from small exhaustive datasets that drugs can be functionally classified from their pairwise interactions [1, 2, 3]. Here, we investigate whether this functional classification can be inferred from our stochastic block model approach, and whether one can do this, not only with small exhaustive datasets, but also with large, non-exhaustive datasets.

In particular, we consider the co-classification probability of pairs of drugs, that is, the probability that two drugs belong to the same group (see Section 2). First, we study the Cokol *et al.* and the Yeh *et al.* datasets (Fig. 5A and C). In both cases, most drugs are consistently classified with a few others indicating that well-defined groups do exist. Moreover, these groups correspond to mechanisms of action. For the Yeh dataset, drugs are consistently grouped with other drugs with the same mechanism of action, with the only exceptions of CHL (which is classified with drugs that target the 50S ribosomal subunit, instead of the 30S subunit) and NAL (which targets DNA gyrase and is not classified with the other two drugs that have the same mechanism of action). A close inspection of the interaction matrix shown in Fig. 5D reveals that CHL and NAL actually have an interaction pattern different to that shown by the other drugs in their putative groups (30S ribosomal subunit and DNA gyrase, respectively) which explain these discrepancies in group assignment. Specifically, we observe that interaction between drugs within the 30S ribosomal are either synergistic or additive, while CHL has antagonistic interactions with drugs in this group. Similarly, interactions between drugs within the DNA gyrase group are synergistic, while NAL has additive interactions with drugs in this group.

Most drugs in the Cokol dataset were selected precisely because each of them acts on a different target, so the analysis is not as straightforward in this case. Still, the only drugs that

share to some extent a mechanism of action (four drugs that act on ergosterol metabolism and two drugs that act on serine/threonine) are again consistently in the same drug groups.

Once drugs are ordered according to their co-classification probabilities in either the Cokol and Yeh datasets (Fig. 4B and D), drug interactions show clear patterns. For example in the Yeh dataset, most interactions between the group $\{\mathrm{AMK}, \mathrm{STR}, \mathrm{TOB}\}$ and the group $\{\mathrm{TMP}, \mathrm{SLF}, \mathrm{NIT}, \mathrm{CPR}, \mathrm{L}$[illegible] are synergistic.

In large and incomplete datasets like DrugBank, an exhaustive co-classification analysis in not feasible. However, DrugBank contains information about drug function and behavior, and we use this information to establish if the co-classification probability of two drugs is correlated with their pharmacological similarity. In particular, we focus on drug targets, drug substructures and drug category [4], and measure drug functional similarity in each of these respects using what we call drug overlap (see Section 3).

As we show in Fig. 5E, drug pairs with higher co-classification probability (especially those with co-classification probability above 0.7) are significantly more likely to share targets, substructures and categories. This indicates that, even for large incomplete datasets, our Bayesian approach is indeed identifying sensible groups of drugs. Interestingly, this also indicates that stochastic block models can be used to infer the function of drugs whose mechanisms are not well understood from the pattern of interaction with other drugs.

3 Estimation of the co-classification probability using stochastic block models

Following arguments analogous to those we use to estimate the probability of an interaction type, the probability $p(\sigma_i = \sigma_j | N^O)$ that drugs i and j belong to the same group (or co-

classification probability) is

$$p(\sigma_i = \sigma_j | N^O) = \frac{1}{Z} \sum_P \delta(\sigma_i, \sigma_j) \exp(-H(P)) \ , \tag{1}$$

where $\delta(x, y) = 1$ if $x = y$ and $\delta(x, y) = 0$ otherwise, and $H(P)$ is the same as in Eq. (6) in the main text. As before, this sum can be estimated using the Metropolis algorithm [5, 6, 7].

4 Drug similarity for drugs in the DrugBank

Each drug in the DrugBank database is labeled with terms that identify, among others, its therapeutic categories (category), its targets (target), and its substructures and functional groups (substructure). For example, hydrocodone is labeled with categories narcotics, analgesics and antitussives, among others; targets MOR-1 and DOR-1; and substructures morphians, benzofurans, and naphtalenes, among others.

For a given classification $C \in \{\mathrm{category}, \ \mathrm{target}, \ \mathrm{substructure}\}$, we define the overlap (or functional similarity) o_{ij} between two drugs i and j as the Jaccard index of the corresponding labels

$$o_{ij}^C = \frac{|L_i^C \cap L_j^C|}{|L_i^C \cup L_j^C|} \ , \tag{2}$$

where L_i^C is the set of labels of drug i in classification C. For example, if drug A has category labels $L_A^{\text{category}} = \{\mathrm{narcotics}, \ \mathrm{analgesics}\}$ and drug B has $L_B^{\text{category}} = \{\mathrm{antitussives}, \ \mathrm{analgesics}\}$, then $o_{AB}^{\text{category}} = 1/3$.

References and Notes

[1] Yeh P, Tschumi AI, Kishony R (2006) Functional classification of drugs by properties of their pairwise interactions. Nat Genet 38: 489–494.

[2] Lehár J, Zimmermann GR, Krueger AS, Molnar RA, Ledell JT, et al. (2007) Chemical combination effects predict connectivity in biological systems. Mol Syst Biol 3: 80.

[3] Yeh P, Kishony R (2007) Networks from drug-drug surfaces. Mol Syst Biol 3: 85.

[4] Knox C, Law V, Jewison T, Liu P, Ly S, et al. (2011) Drugbank 3.0: A comprehensive resource for 'omics' research on drugs. Nucleic Acids Res 39: D1035–D1041.

[5] Metropolis N, Rosenbluth AW, Rosenbluth MN, Teller AH, Teller E (1953) Equation of state calculations by fast computing machines. J Chem Phys 21: 1087–1092.

[6] Guimerà R, Sales-Pardo M (2009) Missing and spurious interactions and the reconstruction of complex networks. Proc Natl Acad Sci U S A 106: 22073–22078.

[7] Guimerà R, Sales-Pardo M (2012) Predicting human preferences using the block structure of complex social networks. PLoS One 7: e44620.
